# The Effectiveness of Online Messages for Promoting Smoking Cessation Resources: Predicting Nationwide Campaign Effects From Neural Responses in the EX Campaign

**DOI:** 10.3389/fnhum.2020.565772

**Published:** 2020-09-25

**Authors:** Ralf Schmälzle, Nicole Cooper, Matthew Brook O’Donnell, Steven Tompson, Sangil Lee, Jennifer Cantrell, Jean M. Vettel, Emily B. Falk

**Affiliations:** ^1^Department of Communication, College of Communication Arts and Sciences, Michigan State University, East Lansing, MI, United States; ^2^Annenberg School for Communication, University of Pennsylvania, Philadelphia, PA, United States; ^3^U.S. Army Research Laboratory, Aberdeen Proving Ground, Adelphi, MD, United States; ^4^Department of Bioengineering, University of Pennsylvania, Philadelphia, PA, United States; ^5^Department of Psychology, University of Pennsylvania, Philadelphia, PA, United States; ^6^New York University School of Global Public Health, New York, NY, United States; ^7^Department of Psychological and Brain Sciences, University of California, Santa Barbara, Santa Barbara, CA, United States; ^8^Wharton Marketing Department, University of Pennsylvania, Philadelphia, PA, United States

**Keywords:** fMRI, health communication, smoking, banner ads, advertising, click-through rate

## Abstract

What are the key ingredients that make some persuasive messages resonate with audiences and elicit action, while others fail? Billions of dollars per year are put towards changing human behavior, but it is difficult to know which messages will be the most persuasive in the field. By combining novel neuroimaging techniques and large-scale online data, we examine the role of key health communication variables relevant to motivating action at scale. We exposed a sample of smokers to anti-smoking web-banner messages from a real-world campaign while measuring message-evoked brain response patterns *via* fMRI, and we also obtained subjective evaluations of each banner. Neural indices were derived based on: (i) message-evoked activity in specific brain regions; and (ii) spatially distributed response patterns, both selected based on prior research and theoretical considerations. Next, we connected the neural and subjective data with an independent, objective outcome of message success, which is the per-banner click-through rate in the real-world campaign. Results show that messages evoking brain responses more similar to signatures of negative emotion and vividness had lower online click-through-rates. This strategy helps to connect and integrate the rapidly growing body of knowledge about brain function with formative research and outcome evaluation of health campaigns, and could ultimately further disease prevention efforts.

## Introduction

Mass media health campaigns are a key component of public health promotion (Wakefield et al., 2010), and the internet has become one of the most prominent channels for dissemination of health campaign messages (Shi et al., 2018). The need to maximize the effectiveness of health messaging is clear given the enormous burden associated with preventable diseases (Mokdad et al., 2004) and the need for efficiency in choosing messages. In the case of tobacco, for example, prevention efforts have only about 5% of the budget available to tobacco companies (Farrelly et al., 2003; CDC/CDCTobaccoFree, 2019). Despite important progress (Cho, 2011; Kim and Cappella, 2019; Sutton et al., 2019), the creation and selection of campaign messages remain a mix of art and science (Noar, 2011; Rice and Atkin, 2012). A deeper understanding of the factors associated with message success is critical for designing more efficient and effective health campaigns.

Neuroimaging is one means of capturing message-evoked responses and improving prediction of health message effectiveness (Falk, 2010; Falk and Scholz, 2018; Schmälzle et al., 2017). A growing body of neuroimaging work has demonstrated that message-evoked brain responses can predict key communication outcomes, such as changes in individuals’ health behavior (Chua et al., 2011; Falk et al., 2011, 2015a; Wang et al., 2015; Riddle et al., 2016; Vezich et al., 2016; Cooper et al., 2017; Zelle et al., 2017; Kang et al., 2018), as well as large-scale outcomes like calls to smoking quitlines (Falk et al., 2015b, 2016). Methodologically, the most widely used approach to capture message-evoked brain responses has relied on assessing the average signal in specific brain regions (Falk and Scholz, 2018). These approaches have primarily focused on the role of regions broadly implicated in positive valuation and personal relevance, such as the nucleus accumbens (NAcc; Knutson et al., 2014; Genevsky and Knutson, 2015; Genevsky et al., 2017; Scholz et al., 2017) and the ventromedial prefrontal cortex (vmPFC; Falk et al., 2012; Venkatraman et al., 2015; Scholz et al., 2017, 2019; Doré et al., 2019a).

Novel neuroimaging methods suggest that using the spatial pattern of activation across the whole brain can carry important information beyond univariate (regional) activity and can improve the prediction of important outcomes. For example, brain activity “signature maps” incorporate spatial information about patterns of activity (or signatures) throughout the whole brain, and can accurately predict responses such as the strength of negative emotion evoked by effective images as in the Picture induced negative emotion signature (*PINES*, Chang et al., 2015), or craving in the context of food choice (Cosme et al., 2020), as well as message-relevant outcomes (Kaplan et al., 2015; Doré et al., 2019b). Comparable signature maps have also been trained for several other domains (Wager et al., 2013; Eisenbarth et al., 2016), including positive emotion and vividness during future-oriented scenario thinking (Lee et al., 2020), among other domains. These signatures have the potential for wide application; that is, they could aid the selection of messages that can elicit brain activity captured by these signature maps.

Here, we test the utility of multivariate brain activity signature maps related to selected key theoretical constructs in predicting health campaign outcomes. Specifically, we focus on neural signatures of negative emotion, positive emotion, and message vividness. Within the health communication literature, negative emotions, such as fear, have been studied extensively (Rogers, 1975; Witte and Allen, 2000; Tannenbaum et al., 2015). For example, highlighting the risks of continuing a behavior (like smoking) can motivate change (Rogers, 1975; Witte, 1992; Renner and Schwarzer, 2003). On the other hand, inducing negative emotions about the process of behavior change (e.g., highlighting the difficulty of change) can also discourage change (Witte, 1994; Maloney et al., 2011; Ruiter et al., 2014).

A second concept, particularly prominent in the social marketing literature (Lee and Kotler, 2011), is positive emotion. Positive emotions are related to the activation of the appetitive motivational system (Elliot, 2013; Lang and Bradley, 2013), and messages that convey the positive value of health behavior change should promote action (Myrick, 2015; Nabi, 2015; Guan and Monahan, 2017), as shown by the success of commercial advertising (Tellis, 2003; Siegel, 2013).

An additional key element of messaging relates to message vividness (Zillmann, 2006; Spence et al., 2017; Blondé and Girandola, 2018; Ophir et al., 2019). On the one hand, highly vivid messages can boost the message’s target emotion and render the described situation more plausible and concrete (D’Argembeau et al., 2008; Ji et al., 2016). For example, in the context of an anti-smoking campaign, vividly imagining the negative consequences of smoking could encourage quitting. However, vivid imagining could also backfire if a message prompted the targeted viewer to vividly imagine how difficult it will be to enact the desired behavior, and instead induce avoidance of behavior change. For example, vividly imagining how hard it will be to quit smoking could reduce the chances that a smoker attempts to quit. In sum, although there is an agreement that emotion and vividness play a role in health message design, they may help or hinder message success.

Using the *“Ex”* tobacco cessation campaign as a real-world test case, we examine whether neuroimaging methods focused on multivariate signatures of key health communication constructs are related to key campaign outcomes. We suggest an approach that helps integrate the health communication literature on emotion and vividness with novel neuroimaging methods. To better understand the processes that predict the success of online messaging, we collected neural data while smokers were exposed to online banner ads promoting smoking cessation. We computed both regional (univariate) and pattern-based (signature map) indices for each banner across this neural sample based on the theoretical considerations outlined above. We used an online sample of smokers exposed to the same banner ads to obtain subjective evaluations about theorized constructs (i.e., negative and positive emotional responses and vividness), to gain insight into whether multivariate brain indices concur with subjective evaluations. We find evidence, from neural and subjective data, that in the context of this campaign, negative emotion and vividness were negatively related to online ad success. Further, we find that although neural and survey data are positively related, prediction of online ad success is improved when using both sources of information, suggesting that theoretically-motivated neural signature maps could ultimately aid in the design and selection of effective messaging in conjunction with more traditional survey-based methods.

## Materials and Methods

This study brings together data from three separate sources: (1) Online banner messages and corresponding click-through-rates from the Truth Initiative’s *Ex* smoking cessation campaign; (2) neural data recorded while a group of smokers was exposed to the *Ex* banners; and (3) subjective evaluations of the *Ex* banners obtained from an online test audience of smokers. Below, we provide methodological details on each data source.

### The Truth Initiative’s Ex Campaign

#### Banner Messages

Twenty-three banner messages, or ads, aired online as part of the *Ex* campaign, run by the Truth Initiative (formerly known as the American Legacy Foundation). The *Ex* campaign launched nationally on March 31, 2008, and continues to run digitally^1^. The banners were professionally produced and shared common design elements. All messages are characterized by an empathetic, smoker-to-smoker tone, with emphasis placed on disassociating smoking from common activities that may function as smoking triggers, such as drinking coffee, drinking alcohol, driving, or stressful situations (see Supplementary Figure S1 for examples). The animated banner messages contained text that empathized with smokers’ needs when quitting or struggles to overcome a specific smoking trigger, and some presented cartoon animations illustrating trigger situations. None contained sound, but some text was present on each. Each banner message ended promoting the EX campaign’s resources for smokers with a variant of the slogan “*Re-learn life without cigarettes at BecomeanEx.org*”, which viewers could click on to be brought to the campaign website for further information and quit smoking resources. The ads averaged 17.7 s in duration (*range* 13.9–30 s, *SD* = 3.9 s).

#### Population-Level Click-Through Rates (CTR)

The 23 banner messages were displayed nationally on websites appealing to the target audience of adult smokers. Viewing metrics were collected by the Truth Initiative, and the response rates described here were collected between January and August 2012. In this period, the banners had an average of 5,895,553 viewings (135,597,715 total across the 23 banners) and received an average of 9,936 clicks (228,517 total across the 23 banners). We computed the CTR for each banner as the number of viewers who clicked on the banner divided by the total viewers exposed to that banner. The average CTR aggregating across banners was 0.21%, and the variability of CTR across banners was quite large, ranging from 0.08–0.57%. One banner message received many fewer impressions than the other banners (9,545 impressions, compared to a range of 1.1–12.6 million impressions in all other banners); this banner was excluded from analysis due to this discrepancy. The CTR scores for individual banners were used to rank their effectiveness within the campaign.

### Brain Responses: Neuroimaging Sample

#### Participants

Fifty smokers participated in the fMRI component of the study. Participants were recruited from the general population using Craigslist and UMClinicalStudies. Interested participants completed an eligibility screening phone call. To participate in the study, participants had to report smoking at least five cigarettes per day for the past month, have been a smoker for at least 12 months, be between the ages of 18 and 65, and meet standard fMRI eligibility criteria. All participants provided informed consent following the procedures of the Institutional Review Board of the University of Michigan. Three participants were excluded due to neurological abnormalities and technical issues. Five participants were excluded due to excessive head motion, at a threshold of greater than 3.5 mm displacement. This resulted in a final sample of 42 participants (24 males, 18 females, *mean age* = 32 years, *SD* = 13 years, *range* 19–64 years). Participants smoked an average of 13 (SD = 7) cigarettes per day.

#### Procedure

Participants watched and rated 23 animated banner messages while undergoing fMRI. Banners were presented in random order across participants. Immediately following each ad, participants were presented with a response screen with the statement “*This makes me want to quit*” and a five point rating scale (*1 = definitely does not, 2 = does not, 3 = neutral, 4 = does, 5 = definitely does*). They were allowed 4 s on the response screen, which was followed by fixation with a jittered ITI (*mean* = 4.1 s, *range* 3.1–7.5 s, *SD* = 1.1).

#### MRI Image Acquisition and Preprocessing

Neuroimaging data were acquired using a three Tesla GE Signa MRI scanner. One functional run of the banner messages task (304 volumes total) was acquired at the end of the scan session for each participant, preceded by other tasks that are not the focus of the current investigation. Functional images were recorded using a reverse spiral sequence (*TR* = 2,000 ms, *TE* = 30 ms, *flip angle* = 90°, 43 axial slices, *FOV* = 220 mm, *slice thickness* = 3 mm; *voxel size* = 3.44 × 3.44 × 3.0 mm). We also acquired in-plane T1-weighted images (43 slices; *slice thickness* = 3 mm; *voxel size* = 0.86 × 0.86 × 3.0 mm) and high-resolution T1-weighted images (SPGR; 124 slices; *slice thickness* = 1.02 × 1.02 × 1.2 mm) for use in coregistration and normalization.

Functional data were pre-processed and analyzed using Statistical Parametric Mapping (SPM8, Welcome Department of Cognitive Neurology, Institute of Neurology, London, UK), AFNI, and Python. We utilized analysis and visualization tools from the NiBabel, Nilearn, and Seaborn packages (Abraham et al., 2014; Waskom et al., 2014). To allow for the stabilization of the BOLD signal, the first five volumes (10 s) of each run were discarded before analysis. Functional images were despiked using the 3d Despike program as implemented in the AFNI toolbox, corrected for differences in the time of slice acquisition using sinc interpolation (the first slice served as the reference slice), and spatially realigned to the first functional image. We then co-registered the functional and structural images using a two-stage procedure. First, in-plane T1 images were registered to the mean functional image. Next, high-resolution T1 images were registered to the in-plane image. After coregistration, high-resolution structural images were skull-stripped using the VBM8 toolbox for SPM^2^ and then normalized to the skull-stripped MNI template provided by FSL. Finally, functional images were smoothed using a Gaussian kernel (8 mm FWHM) for the activation analysis only (pattern-based analyses were computed using the same pipeline except for the smoothing step). The fMRI data were modeled for each participant using fixed-effects models within the general linear model as implemented in SPM8. The six rigid-body translation and rotation parameters derived from spatial realignment were also included as nuisance regressors in the first level models. Data were high-pass filtered with a cutoff of 128 s.

#### Item-Wise Modeling of Responses Towards Individual Banners

To assess the neural response to each ad (in service of computing a per-item response to each ad across the sample; see analysis strategy below), we created an item-wise first-level model in which each banner was modeled in a task regressor separate from a regressor representing all other banners (Mumford et al., 2014). The response periods were modeled in a single boxcar regressor, and fixation rest-periods constituted an implicit baseline. Neural responses to each banner were extracted by iterating over this procedure for all banners for each participant, and then averaging across all 42 participants to obtain a composite beta-map for each banner that served as the basis for extracting univariate or multivariate metrics. See Supplementary Figure S2 for group-averaged neural responses to the banner messages.

#### Region of Interest Analysis

We examined the neural response to each banner in selected brain regions of interest. Specifically, we extracted the average regional activity from the *ventromedial prefrontal cortex* and the *ventral striatum* based on meta-analyses of the valuation system (Bartra et al., 2013). Following Knutson et al. (2014), we computed indices for *positive* and *negative emotional arousal* based on formulae that combine the regional activity of the anterior insula, the ventral striatum, and the anterior cingulate cortex. The region of interest values for each banner was rank-ordered for analyses below.

#### Multivariate Similarity Analysis

Having derived univariate metrics for each banner, we proceeded to multivariate analyses, which focus on whole-brain spatial patterns of banner-evoked brain activity. The *PINES* (picture-induced negative emotion signature) map was obtained from Chang et al. (2015), and the *positive emotion* and *vividness* maps from Lee et al. (2020; see also Supplementary Materials). The brain map of average activation across participants for each banner was then compared to the signature maps of interest for *negative emotion* (*PINES*), *positive emotion*, and *vividness*, respectively, by assessing the spatial correlation of the banner-wise maps and the signature map. The resulting values for each banner were rank-ordered and thus provide a non-parametric measure of fit between the signature map and the neural response to each banner within our sample of viewers.

### Subjective Evaluations: Online Sample

#### Participants

We recruited 50 smokers using Amazon’s Mechanical Turk based on criteria that matched the target audience of the campaign and the fMRI sample (i.e., smoking at least five cigarettes per day for the past month, have been a smoker for at least 12 months, be between the ages of 18 and 65). The study was conducted following the procedures of the Institutional Review Board of the University of Pennsylvania. The final sample consisted of 44 raters after the exclusion of six participants who failed on an attention check (22 males, 22 females, *mean age* = 37 years, *SD* = 9 years, *range* 23–57 years). Participants smoked an average of 15 (*SD* = 6) cigarettes per day.

#### Subjective Evaluation Procedure

As in the fMRI study, each participant viewed each banner message. After viewing each message, participants were asked to evaluate the extent to which the ad generated negative and positive emotion (“*This ad made me feel negative*” and “*This ad made me feel positive*”); how vividly the ad made them imagine scenarios (“*This ad made me vividly imagine reasons not to quit smoking*” and “*This ad made me vividly imagine reasons to quit smoking*”; see Supplementary Materials for results related to the latter question item); and the ad’s effect on their motivation to quit smoking (“*This makes me want to quit*”). All responses were made on a five point scale from “*strongly disagree*”—“*strongly agree*.” Responses were averaged across participants on each dimension for each banner ad. These averages were then ranked within each dimension.

### Analysis

**Figure 1:**
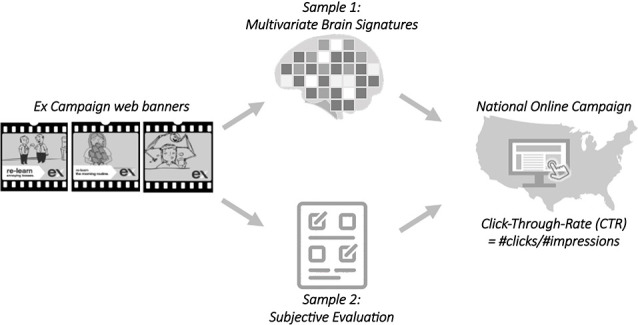
A set of 23 web-banners from the Legacy EX campaign served as stimuli. When these banners aired online, their page impressions and generated clicks were tracked, providing a click-through rate metric for each banner. In the fMRI study, the same banners were shown to a sample of 42 smokers and the pattern of brain activity evoked by each banner message was compared to theoretically-motivated neural signature maps from the fMRI literature. In a parallel online study, we obtained subjective evaluations of banner-evoked negative emotion, positive emotion, and vividness from a different group of 44 smokers. We then tested whether the brain-derived measures and subjective reports for each banner ad predict online click-through rates.

We examined the relationships between online banner ad success (click-through rates—*CTRs*), the neural responses to each banner ad within a test audience, and subjective evaluations of each banner obtained from an online test audience. Thus, the unit of analysis is the individual banner ads from the EX campaign and the brain and subjective responses are collapsed across individuals in each sample to derive a group-level metric for each banner. See Figure 1 for a schematic overview of the analysis approach.

We first test the predictive capacity of univariate regions of interest used in previous work predicting message effectiveness, followed by tests of the multivariate maps of negative emotion, positive emotion, and vividness. Next, we test whether subjective evaluations, both from the online sample and the scanned participants, predict click through rates. Finally, we examine neural metrics and subjective evaluations in combined regression models to test whether having both sources of information improves the prediction of online banner message success (*CTRs*). In all correlation tests and regression models, we use the ranked measures described in each section above (results with unranked measures are in Supplementary Material). Ranks provide a non-parametric and robust way to compare variables across varied datasets. The extension of multiple regression on ranks has been studied by Iman and Conover (1979) and a similar approach has been used in previous work (Weber et al., 2015; Falk et al., 2016). In addition to the benefits of non-parametric analysis, ranked analyses are also desirable for practitioners who strive to select the best messages.

## Results

### Brain Measures Predict Online Banner Ad Success

To test the importance of three specific constructs^3^—negative emotion, positive emotion, and vividness—in the online success of the anti-smoking banner ads used in this campaign, we examined the average brain responses to the banners within key brain regions involved in these processes as well as recently developed multivariate patterns and related them to banner click-through-rates (*CTR*).

We began by testing for relationships between *CTR* and the per-banner neural indices derived from average brain activity in regions associated with the relevant constructs (negative and positive emotion) and in regions associated with message success in prior work. We found that per-banner brain activity in regions associated with negative emotional arousal (Knutson et al., 2014) was negatively related to *CTR* (*r* = −0.44, *p* = 0.04). In other words, banners that prompted the strongest response in these regions were those that received the least click-throughs. No significant relations were observed for the positive emotional arousal index (*r* = −0.01, *p* = 0.96) or for regions associated with positive valuation (*VS*: *r* = −0.14, *p* = 0.52; *vmPFC*: *r* = −0.05, *p* = 0.83).

**Figure 2:**
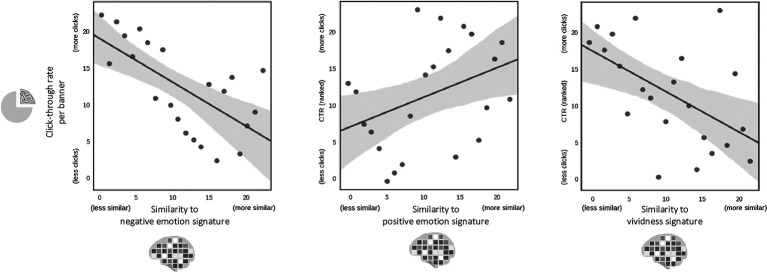
The degree to which the pattern of brain activity for each banner matches neural signature maps for negative and positive emotion, as well as vividness, is negatively linked to the online success (click-through-rates) of the same banners. Banners that prompt brain activity patterns that better match negative emotion and vividness signatures tend to have lower online success (i.e., lower click-through rates).

Next, we computed the degree of similarity between the average whole-brain activation map for each banner and the brain signature maps for each of the three theoretically-motivated health communication constructs. The brain signature maps have been previously validated for their respective purpose (i.e., to predict negative or positive emotion and vividness, respectively) in independent datasets. We find that banners with higher similarity to the Picture-Induced-Negative-Emotion-Signature (*PINES*) have lower *CTRs* (*r* = −0.66, *p* = 0.0008, Figure 2, left)^4^. In other words, the more a banner evoked a brain response resembling the pattern of the negative emotion signature, the fewer clicks it generated in the real-world online campaign. We found that similarity to the positive emotion signature was marginally positive, though not significantly, associated with *CTRs* (*r* = 0.38, *p* = 0.082, Figure 2, middle). Banners with higher similarity to the vividness signature generated lower *CTRs* in the real-world campaign (*r* = −0.53, *p* = 0.011, Figure 2, right). That is, when the evoked brain response was more similar to the vivid brain signature, viewers were less likely to click through to online resources to quit smoking. These results suggest that banners that more closely resemble the brain maps for negative emotion and vividness were less successful online.

### Subjective Evaluations Predict Online Banner Ad Success

**Figure 3:**
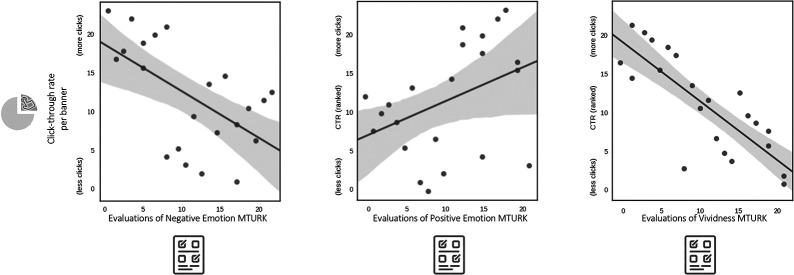
Linking subjective reports of negative emotion, positive emotion, and vividness per banner to the population-level click-through rate generated by the same banners.

Next, we examined the subjective evaluations collected from our online sample of MTurk smokers. Participants rated the extent to which each ad generated negative or positive emotion, and how vividly the ad made them imagine reasons not to quit smoking. There was a negative relationship between negative emotion ratings and *CTR* (*r* = −0.58, *p* = 0.005; Figure 3, left), such that banners eliciting higher levels of negative emotion had lower *CTRs*. Positive emotion ratings were positively, but only marginally associated with *CTR* (*r* = 0.39, *p* = 0.07; Figure 3, middle). Finally, vividness showed a strong negative relationship to *CTR* (*r* = −0.81; *p* = 0.000006; Figure 3, right), such that banners eliciting higher levels of vividness had lower *CTRs*. These results parallel the findings described above for the multivariate signature maps of emotion and vividness.

### Brain Measures Improve Predictions of Online Banner Ad Success

Next, we tested whether the neural metrics provide additional explanatory power when combined in multiple regression models with subjective evaluations. To do this, we compared the *R*^2^ values for models with subjective evaluations alone to *R*^2^ values for models with brain similarity metrics combined with subjective evaluations.

We first consider negative emotion. The addition of the brain similarity metric to a model containing subjective evaluations alone increased the explained variance in *CTR* from *R*^2^ = 0.33 to *R*^2^ = 0.45, which is a marginal improvement in model fit (*F*_(1,19)_ = 4.1, *p* = 0.057; see Supplementary Figure S3 for correlations between all brain and subjective metrics). We find a similar result for vividness. The addition of the brain similarity metric to a model containing subjective evaluations increased the explained variance in *CTR* from *R*^2^ = 0.65 to *R*^2^ = 0.73, which is a significant improvement in model fit (*F*_(1,19)_ = 5.71, *p* = 0.027)^5^. The addition of the positive emotion brain similarity metric to a model containing subjective evaluations of positive emotion did not significantly increase the explained variance in *CTR* (*R*^2^ = 0.16 to *R*^2^ = 0.23; model comparison *F*_(1,19)_ = 1.87, *p* = 0.19), though the effect size was comparable to the other construct maps.

Thus, although the brain similarity metrics and subjective evaluations are correlated with each other (Supplementary Figure S3), they are not fully redundant. Practically, having information from both sources is useful for prediction, as the combined models are a significantly better fit than models with subjective evaluations alone.

## Discussion

We recorded neural responses and obtained subjective evaluations from two samples of viewers exposed to anti-smoking banner messages from the Truth Initiative’s online *EX* campaign, and we linked these data with large-scale metrics of effectiveness for the same banners at scale. We find that multivariate brain maps assessing negative emotion and vividness in a small group of smokers who underwent brain imaging were strongly and negatively associated with the success of the same banner messages online, with over 135 million viewings during the nationwide online campaign. Further, we find that these multivariate brain metrics improve predictions of message success relative to regional activation in select brain areas of interest, and relative to exclusively using self-reported evaluations of the ads made by a separate sample of smokers. These data support the utility of neuroimaging techniques in understanding and forecasting public health campaign effectiveness (Falk and Scholz, 2018).

Although the *EX* campaign was highly successful overall (Vallone et al., 2010, 2011), there was variability in the online success of individual banner messages. Specifically, our results show that banner messages which elicited a brain response more similar to a negative emotion signature (Chang et al., 2015) were less successful in attracting clicks from viewers in this context. These results are in line with the notion that increased negative emotion can detract from the effectiveness of messages (Peters et al., 2013; Ruiter et al., 2014), and recent findings showing that although some kinds of emotion (e.g., fear; Tannenbaum et al., 2015) can improve campaign outcomes, strong negative emotion in smoking cessation messages does not always increase the call-volume to quitlines (Farrelly et al., 2011). As such, people may be less likely to take action towards quitting when messages prompt negative emotion or are framed in ways that highlight the problems with past behavior (Rothman and Salovey, 1997). This could be particularly true in contexts where viewers are likely to react defensively, such as smokers viewing anti-smoking messages (Sweeney and Moyer, 2015; Memish et al., 2017) or when vividly imagining how difficult it might be to quit. Furthermore, particularly in the new media environment, banner messages are presented alongside other information that they compete with for the viewer’s limited attentional resources, and the self-paced and easily terminated nature of browsing behavior might promote avoidance of information that elicits negative emotion.

We also examined the role of vividness in message effectiveness and found that brain responses more consistent with high levels of vividness were associated with lower population level click through rates. As is the case for negative emotion, prior research has shown competing effects of vividness (Taylor and Thompson, 1982; Collins et al., 1988; Smith and Shaffer, 2000). For example, it seems intuitive that higher message vividness—often in tandem with negative emotion—could improve message effectiveness, for example, if people vividly imagine the consequences of their behaviors. However, other reports find that high vividness can be detrimental if creative special effects overshadow the message’s main point (Smith and Shaffer, 2000; Guadagno et al., 2011). Another explanation is that vividly imagining how hard it will be to quit smoking may demotivate the approach behavior of clicking on the link to obtain more information about quitting. Indeed, the subjective data from MTurk raters support this idea, as the ratings of banner-elicited negative thoughts (“*This ad made me vividly imagine reasons not to quit smoking*”) were very strongly anti-correlated with message success (*r* = −0.81). Future work may test this possibility by, for example, using think-aloud protocols to tap into participants’ thought processes after message exposure (Cacioppo et al., 1997; Pei et al., 2019).

Finally, banner messages eliciting a brain response more similar to the positive emotion signature were marginally more successful in attracting clicks online (*r* = 0.38), but the relationship was statistically not significant. The same pattern emerged for the subjective evaluations, i.e., a marginally positive relationship between the per-banner ratings of positive emotion and click-through rate that was not statistically significant (*r* = 0.37). Of note, the brain-based and the subjective measures also correlated positively (*r* = 0.29), but again not significantly. Work in other contexts, such as predicting crowd-funding outcomes and video virality using brain data, has made a case for positive arousal as a key factor (Genevsky et al., 2017; Tong et al., 2020), but in the current dataset neither the univariate analyses, the multivariate signature, or the subjective evaluations correlated significantly with the click-through measures. One possible explanation is that the relationships would have been significant with a larger number of banner messages, which is a naturally limiting factor in campaigns that use only a small-to-moderate number of messages (here 23 banners). Alternatively, this could suggest that either the context of health prevention messaging or the specific outcome behavior of clicking on an online banner may be important to consider for such brain-behavior analyses.

Concerning the methods used in health prevention neuroimaging, we show that neural signatures developed to detect images-evoked negative emotion (Chang et al., 2015) and neural signatures based on data tracking vividness (Lee et al., 2020; also see Supplementary Materials) were indicative of negative responses to health messaging. This suggests that the brain activity signature maps can be useful outside of the context in which they were directly developed (i.e., maps of basic processes like negative emotion and vividness being successfully applied to predict the success of health messages). We also find that subjective evaluations of the emotions and vividness induced by the banner ads correlate positively with expression of the corresponding signature maps, providing support for our assumption that the signature maps correlate with the constructs for which they were developed—even in very different contexts. That said, although the relationships between subjective evaluations of the ads and multivariate brain metrics to click-through-rates are closely related, information from both sources is useful for the prediction of campaign outcomes. More broadly, this approach offers a principled strategy to integrate neural and subjective data sources.

This demonstration of the viability to use multivariate brain signatures to predict message success also suggests the possibility to train signatures for tapping into sub-aspects of message-induced persuasion. Specifically, while we chose the signatures based on theoretical considerations in the context of health communication, one could attempt to develop new signatures that are directly trained to tap into phenomena of interest rather than using available signatures (Chang et al., 2015; Cosme et al., 2020; Lee et al., 2020). The long-term goal of discovering such markers will be greatly aided by high-quality behavioral outcome metrics, such as large-scale click-through-rates. The click-through rates used to operationalize effectiveness in the current study are an objective behavioral measure of message success at the aggregate level, comparable to audience ratings or sales revenue in other domains, or the persuasion rate more broadly (DellaVigna and Gentzkow, 2010). In terms of the goal of the banners to influence people towards taking the first step towards quitting, i.e., to sign up for quit resources, the click-through rates measured here are a key outcome metric (Rhodes and Ewoldsen, 2013).

Importantly, population-level effects on click-through rates can only emerge if the relevant message—in this case, the banner—can collectively influence a large number of individuals (Imhof et al., 2017, 2020; Grall and Schmälzle, 2020). Our strategy to capture brain activity within a smaller sample of current smokers exposed to the same messages probes this collective-level response to messaging. Moreover, message-evoked brain activity patterns from a relatively small group of individuals can be linked to population-level behavior in response to the banner messages (Knutson and Genevsky, 2018). Going forward, social media-based health communication offers ideal opportunities to measure mass behavior at scale (O’Donnell and Falk, 2015; Tan et al., 2016; Matz et al., 2017) and to jointly examine mechanisms and effects of health prevention messaging.

With this in mind, future research should expand the range of methods used to capture responses to health prevention messages. For instance, Imhof et al. (2017, 2020) used fMRI and EEG methods to measure message-evoked brain responses towards the same set of health messages and demonstrated the possibility of EEG-informed fMRI analyses. Such a strategy seems feasible for the current design and implementing it in future studies promises several insights: if both methods can tap into the relevant message-evoked processes, they can make measurements more robust and provide cross-validation; if, on the other hand, the methods tap into different processes that are relevant for CTR or other outcomes, then it will be important to tease apart which methods are most sensitive for the specific process. The same reasoning also applies to other methods used to study biobehavioral responses to health messages, such as eye-tracking (e.g., Lochbühler et al., 2016), psychophysiological measures, or functional-near-infrared spectroscopy.

Another issue that awaits further research relates to the match between the samples used for message testing and the campaign target audience. The EX campaign was created to help current smokers who are open to quitting and accordingly we recruited only smokers for the fMRI and online studies. However, it will be interesting to examine which specific audience characteristics and individual differences—demographic, psychological, and behavioral—must be matched between the testing samples and the campaign target audience. For instance, it has been shown that brain responses to health risk communication differed between individuals depending on their preexisting level of risk perception (Schmälzle et al., 2013) and that responses to anti-drug messages differed between individuals based on their drug use risk (Huskey et al., 2017). Thus, while it is common wisdom in the social sciences that samples need to be representative of the population to warrant robust inferences, questions remain whether certain responses to messages are obligatory across all receivers and which individual differences must be taken into account. In the current study, the neural and subjective measures obtained for each banner were collapsed across individuals in the fMRI and online samples, respectively, to derive measurements for each banner and relate these to the per-banner CTRs. However, given that individual variability exists in how both the neural and the online samples respond to each message, another question will be how many individuals are needed to obtain robust group-level metrics. Kim and Cappella (2019) suggest that collapsing ratings from about 25 raters per message provides stable ratings when norming health messages for constructs like perceived effectiveness or perceived argument strength. Prior neuroimaging studies aiming to use average brain responses from small groups to predict larger campaign success have used sample sizes between 18 and 47 (Knutson and Genevsky, 2018). Our samples of over 40 participants are within this range, but more work is needed to gauge the individual differences and develop message-testing protocols for neuroimaging that establish how many brain responses are required for optimal prediction.

## Summary and Conclusion

In sum, we bring together perspectives from the neuroscience of emotion and persuasion (Lindquist et al., 2012; Falk and Scholz, 2018) with large-scale public health campaigns (Wakefield et al., 2010; Rice and Atkin, 2012) by linking message-wise brain effects to population-level message effects. We find that multivariate brain signatures of negative emotion and vividness in response to anti-smoking messages in this context were negatively associated with the real-world success of the messages, i.e., the click-through rates generated by individual banner messages. This strategy yields insights into the neurocognitive processes of message reception and behavior change and may thus help to link the rapidly growing body of knowledge about brain function with formative and evaluation research of health campaigns (Noar, 2011; Rice and Atkin, 2012). Integrating functional neuroimaging with large-scale outcome data about campaign effects is a promising avenue for future work that can improve public communication campaigns as well as other forms of information dissemination.

## Data Availability Statement

The raw data supporting the conclusions of this article will be made available by the authors, without undue reservation.

## Ethics Statement

The studies involving human participants were reviewed and approved by Institutional Review Board of the University of Michigan Institutional Review Board of the University of Pennsylvania. The participants provided their written informed consent to participate in this study.

## Author Contributions

RS, NC, MO’D and EF formulated the investigation. RS and NC performed the analysis in discussion with JV and EF. SL generated the valence and vividness signature maps. RS, NC, JV and EF wrote the article. MO’D and ST collected the data and provided analysis tools. Truth Initiative: collected and provided data and analysis tools. All authors contributed to the article and approved the submitted version.

## Conflict of Interest

The authors declare that the research was conducted in the absence of any commercial or financial relationships that could be construed as a potential conflict of interest.

## Footnotes

^1^www.becomeanex.org

^2^http://dbm.neuro.uni-jena.de/vbm

^3^Of note, the responses to the question “This ad makes me motivated to quit smoking” did not significantly predict population-level click-through-rates—neither in the fMRI sample (*r* = −0.01, *n.s*.), nor in the online sample (*r* = −0.09, *n.s*.).

^4^To test whether the negative arousal univariate index and the negative emotion multivariate signature map capture the same variance in *CTR*, we entered both neural metrics into a regression model predicting *CTR*. When these predictors are combined in the same model, the negative emotion brain predictor is significantly related to *CTR* (*t*_(19)_ = −3.71, *p* = 0.001), and negative arousal is not (*t*_(19)_ = −1.95, *p* = 0.067), suggesting that similarity to the multivariate map provides unique information.

^5^Subjective evaluations did not significantly improve model fit beyond what was explained by the neural signature of negative emotion (*R* = 0.44 to *R* = 0.45; model comparison *F*_(1,19)_ = 0.42, *p* = 0.52) or positive emotion (*R* = 0.14 to *R* = 0.23; model comparison *F*_(1,19)_ = 2.18, *p* = 0.16). A combined model with subjective evaluations of both subjective and neural signatures of vividness did improve model fit beyond a model with neural signatures alone (*R* = 0.29 to *R* = 0.73; model comparison *F*_(1,19)_ = 31.2, *p* = 2.2^e-05^).
