# Supplementary material for: The Effectiveness of Online Messages for Promoting Smoking Cessation Resources: Predicting Nationwide Campaign Effects From Neural Responses in the EX Campaign
Source: Front Hum Neurosci. 2020 Sep 25;14:565772. doi: 10.3389/fnhum.2020.565772 (PMC7546826; doi:10.3389/fnhum.2020.565772)

Supplementary Material

# Supplementary Figures and Tables

## Supplementary Figure 1: Still shots of banner ads

***
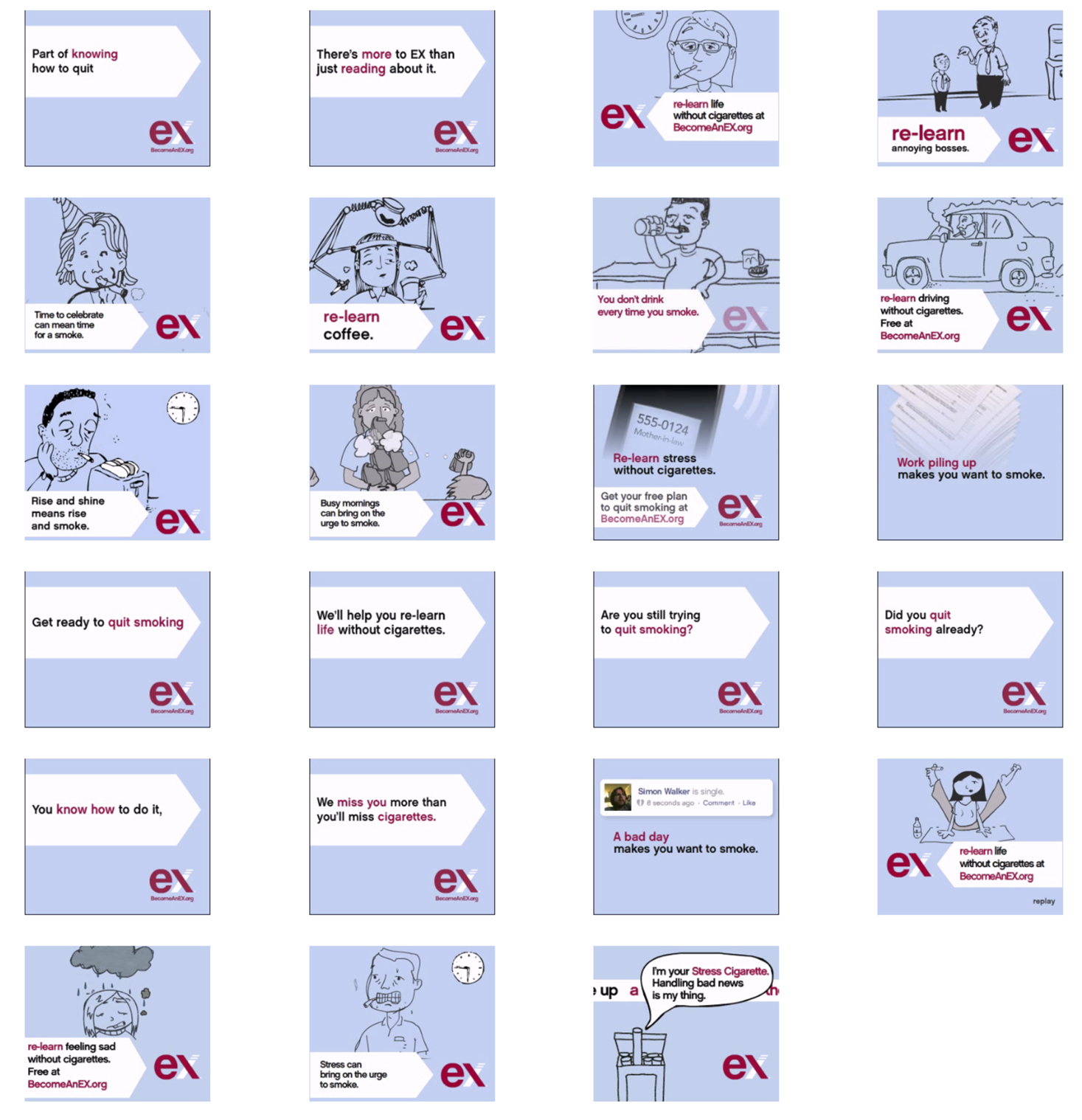
***

## Supplementary Figure 2: Multivariate maps of group-level activation averaged across all banner messages as well as for two exemplar banners. These maps are derived using item-wise GLM-models and are displayed as unthresholded t-maps to illustrate the variability. As expected, this revealed activity in extended visual cortex, parietal and frontal regions encompassing the dorsal attention network, as well as cortical midline structures including the precuneus, anterior cingulate, and medial prefrontal cortex. Slice coordinates: *x = 0, y = 30, z = 20*.

##
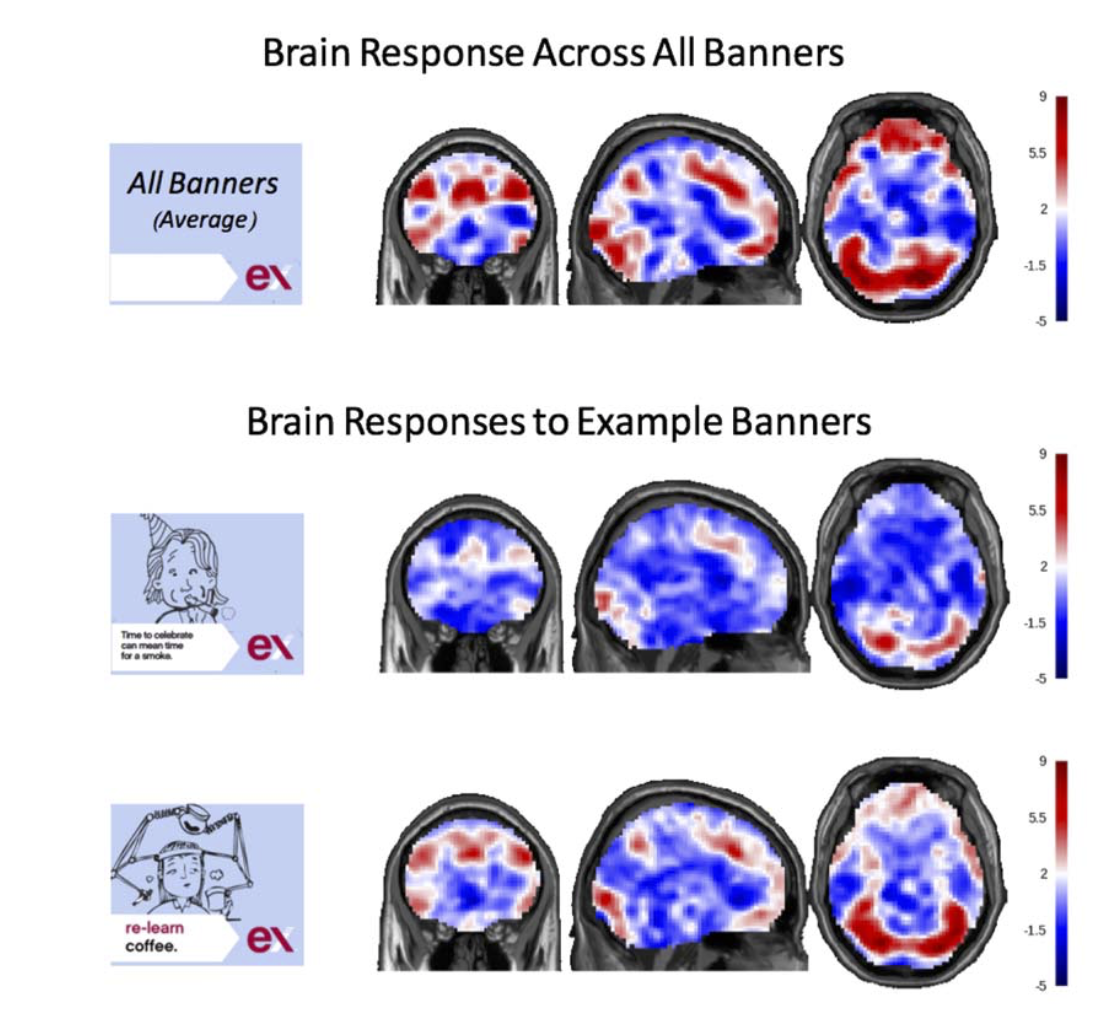


## Supplementary Figure 3: Correlations between measures. Colored cells indicate that the respective measures are significantly related (p<0.05). As in the main text, each variable is rank-ordered prior to correlations. Naming key: Colored cells are p<0.05. U_NegAro = univariate map of negative arousal (Knutson et al., 2014); U_PosAro = univariate map of positive arousal (Knutson et al., 2014; U_VS = univariate average activity in ventral striatum (Bartra et al., 2013); U_VMPFC = univariate average activity in ventromedial prefrontal cortex (Bartra et al., 2013); S_NegEmo = similarity to negative emotion map (PINES; Chang et al); S_PosEmo = similarity to positive emotion map (Lee et al.); S_Vivid = similarity to vividness map (Lee et al.); M_VividQuit = MTurk subjective evaluations of “This ad made me vividly image reasons to quit smoking; MVividNotQuit = MTurk subjective evaluations of ““This ad made me vividly imagine reasons not to quit smoking”; M_NegEmo = MTurk subjective evaluations of negative emotion; M_PosEmo = MTurk subjective evaluations of positive emotion.


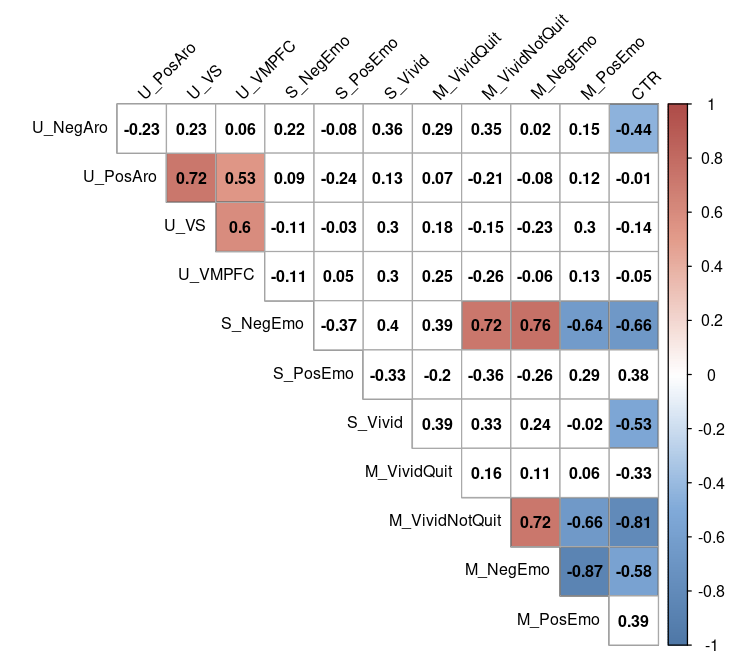


## Supplementary Figure 4: Correlations between measures, unranked. Colored cells indicate that the respective measures are significantly related (p<0.05). Contrary to the main text, variables are NOT rank-ordered. Naming key: U_NegAro = univariate map of negative arousal (Knutson et al., 2014); U_PosAro = univariate map of positive arousal (Knutson et al., 2014; U_VS = univariate average activity in ventral striatum (Bartra et al., 2013); U_VMPFC = univariate average activity in ventromedial prefrontal cortex (Bartra et al., 2013); S_NegEmo = similarity to negative emotion map (PINES; Chang et al); S_PosEmo = similarity to positive emotion map (Lee et al.); S_Vivid = similarity to vividness map (Lee et al.); M_VividQuit = MTurk subjective evaluations of “This ad made me vividly image reasons to quit smoking; MVividNotQuit = MTurk subjective evaluations of ““This ad made me vividly imagine reasons not to quit smoking”; M_NegEmo = MTurk subjective evaluations of negative emotion; M_PosEmo = MTurk subjective evaluations of positive emotion


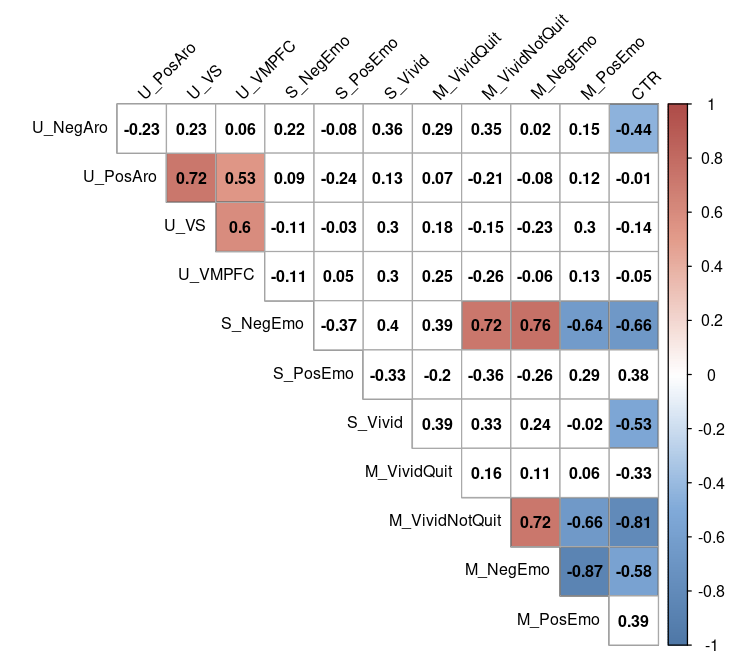


## Supplemental Methods

## 2.1. Signature maps for positive emotion and vividness

# The multivariate signature maps for positive emotion and vividness were created from a dataset by Lee, Parthasarathi & Kable (2020), following the strategy used by Chang et al. (2015) to develop the PINES negative emotions signature. See the paper for full details of participants and the experimental design. In brief, Lee, Parthasarathi & Kable examined neural activity associated with imagining future events (cf. [(D’Argembeau et al., 2008)](https://paperpile.com/c/jw34SZ/Sf9a).) Twenty-four participants underwent fMRI scanning while imagining 64 different future scenarios, in a 2x2 design (positive versus negative valence crossed with high versus low vividness). Participants’ ratings of the scenarios were consistent with the designed condition labels, and condition labels were used in these analyses to train multivariate pattern analysis predictors.

# Specifically, to create whole brain predictors for the different scenario types, Least Absolute Shrinkage and Selection Operator and Principle Components Regression (LASSO-PCR) were used, following the strategy of prior work to derive the PINES signature (Chang, Gianaros, Manuck, Krishnan, & Wager, 2015; Wager, Atlas, Leotti, & Rilling, 2011; Wager et al., 2013). The 24 participants’ data (i.e., 24 participants x 64 trials) first went through principal component decomposition to identify the top 200 components that explained the most variance. These 200 components were then used in a LASSO logistic regression to predict a binary indicator variable for condition labels (coding 1 for positive valence and high vividness, respectively). The best penalty parameter for LASSO was selected by performing a leave-one-subject-out cross validation (24-fold) within the dataset. The resulting coefficients for these 200 principal components were multiplied by their respective components’ brain weight maps and then summed to yield one whole-brain prediction betamap.

To assess out-of-sample prediction accuracies, we performed 24-fold leave-one-subject-out cross validation, training the predictor on data from 23 subjects and testing on the one left-out subject. The 23 subjects’ data (i.e., 23 subjects x 64 trials) in the training fold first went through principal component analysis to identify the top 200 components that explained the most variance. These 200 components were then used in a LASSO logistic regression, for which the best penalty parameter $\lambda$ was selected by performing a secondary leave-one-subject-out cross validation (23-fold) within the training set. The predicted variable in the LASSO logistic regression was either a binary indicator variable for positive valence versus negative valence or a binary indicator for high versus low concreteness. The resulting coefficients for these 200 principal components were multiplied by their respective components’ brain weight maps and then summed to yield one whole-brain prediction betamap. To predict valence or concreteness, we calculated the dot product between the neural predictor of valence or concreteness and the estimated activity brain image from each trial in the testing set, and then added the intercept from the LASSO logistic regression. These scores were then logit transformed to calculate the probability of the trial being high/low concreteness or high/low valence. Balanced prediction accuracy was measured by making predictions based on a threshold of *p* = 0.5. Balanced accuracies were calculated by averaging the accuracy for the two trial types.

The neural signature of valence discriminated positive versus negative imagined events at above chance out-of-sample accuracy (median prediction accuracy = 56%, sign rank test against 50%, *Z* = 4.08, *p* < .001), but did not discriminate imagined events of high versus low concreteness (median prediction accuracy = 50%, Z = 1.28, p > .05). While this neural signature incorporates signals from the entire brain, notably, the most consistent positive weights for valence prediction were in medial prefrontal cortex and posterior cingulate cortex (Figure 1). In contrast, the neural signature of concreteness discriminated imagined events rated high versus low in concreteness (median prediction accuracy = 57%, sign rank test against 50%, *Z* = 2.91, *p* = .004), but not those rated positive versus negative in valence (median prediction accuracy = 52%, sign rank test against 50%, *Z* = 0.98, *p* > .05). Notably, the most consistent positive weights for concreteness prediction were in medial and central orbitofrontal cortex and the hippocampus (Figure 1).

## Supplementary Figure 5: S Signature maps for positive emotion and vividness. Slice coordinates: *x = 0, y = 30, z = -10*.


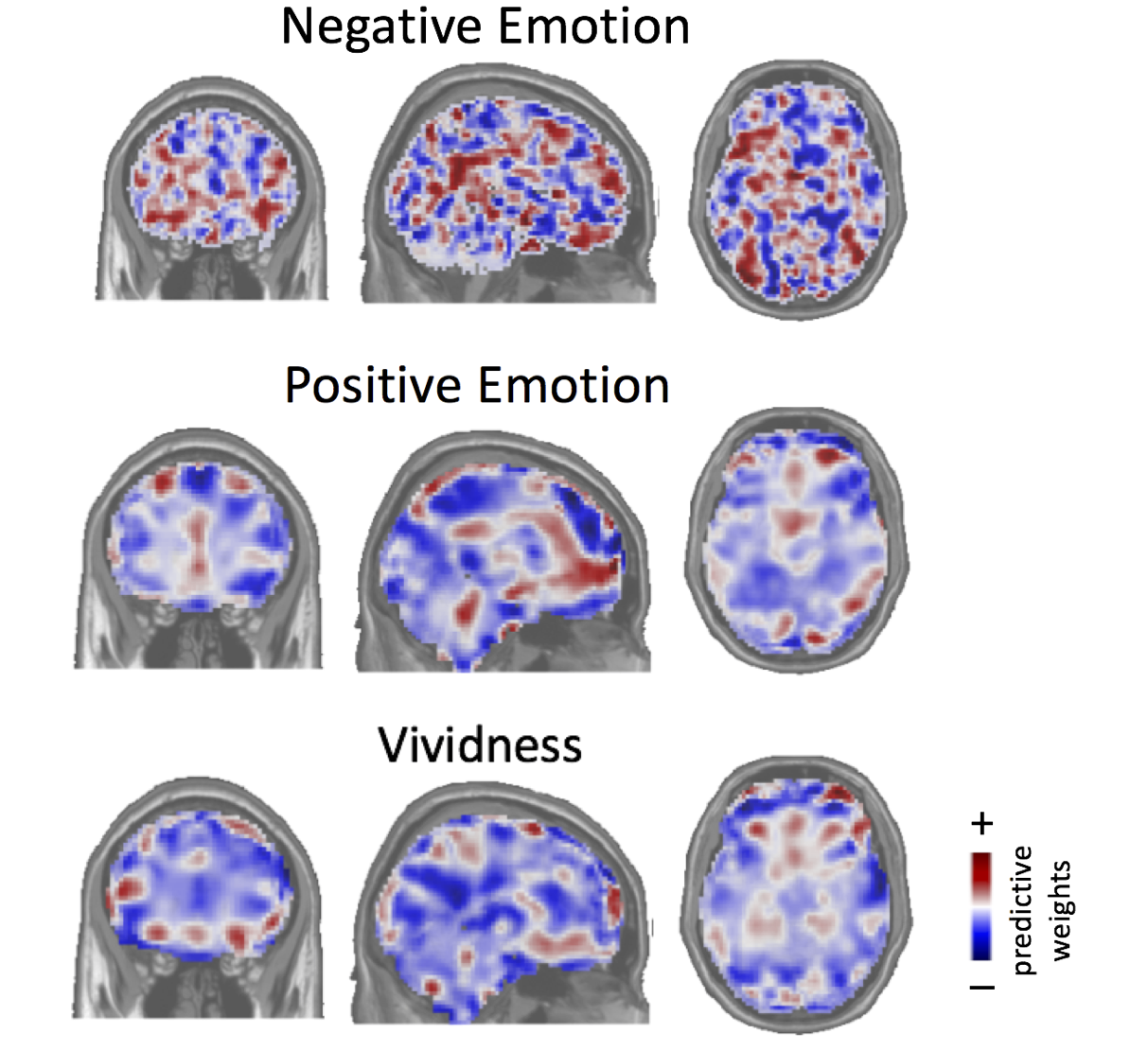

Supplement: Supplementary file 1 [file Table_1.DOCX]
